# Study on the Regulatory Mechanism of the PDK1-Mediated TGF-β/Smad Signaling Pathway in Hypoxia-Induced Yak Lungs

**DOI:** 10.3390/ani14162422

**Published:** 2024-08-21

**Authors:** Yiyang Zhang, Jun Wang, Meng Zhang, Xiaoyun Li, Fan Zhang, Manlin Zhou, Kun Yang, Weiji Chen, Haie Ding, Xiao Tan, Qian Zhang, Zilin Qiao

**Affiliations:** 1 Engineering Research Center of Key Technology and Industrialization of Cell-Based Vaccine, Ministry of Education, Northwest Minzu University, Lanzhou 730030, China; zyy15890182033@163.com (Y.Z.); lixy000920@163.com (X.L.); 15174462439@163.com (F.Z.); 15888533443@163.com (M.Z.); qzl13639315431@163.com (Z.Q.); 2 Gansu Tech Innovation Center of Animal Cell, Biomedical Research Center, Northwest Minzu University, Lanzhou 730030, China; 3 Key Laboratory of Biotechnology and Bioengineering of State Ethnic Affairs Commission, Biomedical Research Center, Northwest Minzu University, Lanzhou 730030, China; 4 Life Science and Engineering College, Northwest Minzu University, Lanzhou 730030, China; wangjun200005@126.com (J.W.); ceisbfai@163.com (M.Z.); cwj20240714@163.com (W.C.); wyyx20230825@126.com (H.D.); wyyx1157@126.com (X.T.); 5 College of Veterinary Medicine, Gansu Agricultural University, Lanzhou 730070, China; zq880204@126.com

**Keywords:** yak, yellow cattle, pulmonary artery smooth muscle cells (PASMCs), hypoxia, pyruvate dehydrogenase kinase 1 (PDK1), TGF-β/Smad signaling pathway

## Abstract

**Simple Summary:**

The lungs are key organs in mammals that shows adaptive changes in response to high altitude, and yaks (*Bos grunniens*) have adapted their lungs well to the hypoxic environment of Tibetan Plateau after a long period of evolution and natural selection. However, the long-term life of lowland cattle on the plateau can cause symptoms such as pulmonary arterial hypertension, wherein the abnormal proliferation of pulmonary vascular smooth muscle cells leads to the remodeling of the pulmonary vasculature, which is the main cause of pulmonary arterial hypertension. The underlying molecular mechanisms of lung adaptation to hypoxia in yaks remain largely unknown. In this study, we constructed stably transfected yak cell lines overexpressing PDK1 via lentiviral transfection, and we cultured yellow cattle and yak PASMCs, as well as PDK1-OEcon/OE, under normoxia and 10% O_2_ to explore the effects of hypoxia on the PDK1 and TGF-β/Smad signaling pathways in yak PASMCs. This will provide theoretical data at the cellular and molecular levels, along with basic information to further elucidate the mechanism of the PDK1-mediated TGF-β/Smad signaling pathway in yak PASMCs induced by hypoxia.

**Abstract:**

The aim of this study was to investigate the effects of hypoxia-induced phenotype, glucose metabolism, ROS levels, and the PDK1-mediated regulation of TGF-β/Smad signaling in yellow cattles, yaks, and those overexpressing PDK1 PASMCs using growth curves, flow cytometry, scratch experiments, glucose and lactic acid assays, RT-qPCR, and Western blotting. The results showed that hypoxia significantly promoted proliferation, migration, antiapoptosis, ROS levels, glucose consumption, and lactate production in yellow cattle PASMCs (*p* < 0.05), and the cells were dedifferentiated from the contractile phenotype; conversely, hypoxia had no significant effect on yak PASMCs (*p* > 0.05). PDK1 overexpression significantly promoted proliferation, antiapoptosis, glucose consumption, and lactate production in yak PASMCs under normoxia and hypoxia (*p* < 0.05), decreased their migration levels under hypoxia (*p* < 0.05), and dedifferentiated the contractile phenotype of the cells. Overexpression of PDK1 in yak PASMCs is detrimental to their adaptation to hypoxic environments. Yak PASMCs adapted to the effects of hypoxia on lung tissue by downregulating the expression of genes related to the PDK1 and TGF-β/Smad signaling pathways. Taken together, the regulation of PDK1-mediated TGF-β/Smad signaling may be involved in the process of yaks’ adaptation to the hypoxic environment of the plateau, reflecting the good adaptive ability of yaks. The present study provides basic information to further elucidate the mechanism of PDK1-mediated TGF-β/Smad signaling induced by hypoxia in the lungs of yaks, as well as target genes for the treatment of plateau diseases in humans and animals.

## 1. Introduction

The plateau region is characterized by unique environmental conditions, including intense ultraviolet radiation, short grazing seasons, low temperatures, and hypoxia. Yaks are endemic to the Tibetan Plateau and have developed unique adaptations such as large lung capacity, and efficient foraging and metabolism [1,2]. Yaks’ adaptations to this hostile environment are closely linked to their lungs [3,4]. Plains mammals are prone to pathologies such as pulmonary hypertension and right-heart hypertrophy after migrating to the hypoxic environment of the plateau, which, after prolonged feeding, result in pulmonary vascular diameter changes and remodeling in the early stages of the pulmonary circulation. The abnormal proliferation of pulmonary artery smooth muscle cells (PASMCs) is a major cause of pulmonary vascular remodeling [5,6] and leads to the development of alpine diseases such as pulmonary hypertension, a devastating cardiopulmonary disease in plains mammals. These adaptations are passed on to offspring, making the yak an ideal species to study how organisms adapt to high-altitude, hypoxic environments [7,8].

Vascular remodeling in the lungs is mainly caused by abnormal proliferation and migration of PASMCs, along with fibrosis of the inner and outer vascular membranes [9]. Since the vascular mesentery composed of smooth muscle cells has been the focus of vascular remodeling studies, the role of phenotypic transformation of smooth muscle cells in vascular remodeling has also attracted much attention. It was found that hypoxia induced a decrease in contractile markers of PASMCs in rats, accompanied by an increase in the production of reactive oxygen species and hydrogen peroxide, resulting in the transformation of PASMCs from contractile to synthetic [10]. It is evident that hypoxia-induced oxidative stress is closely related to the phenotypic transformation of PASMCs. Pyruvate dehydrogenase kinase (PDK) is a key enzyme that reduces pyruvate oxidation in the mitochondria and increases the conversion of pyruvate to lactate in the cytoplasm during hypoxia [11]. PDK1 may be associated with pulmonary vascular remodeling and may be involved in regulating the vascular remodeling process by modulating the pathways of cell proliferation, apoptosis, metabolism, and inflammation. Meanwhile, PDK1 activation inhibits pyruvate dehydrogenase (PDH) activity, leading to a compensatory increase in fatty acid metabolism and oxidation, along with elevated levels of reactive oxygen species [12,13], further impairing mitochondrial function and accelerating the hypoxia-induced transformation of the PASMC phenotype [14]. This suggests that PDK1 is highly involved in the proliferative phenotype of PASMCs, leading to impaired mitochondrial function and altered cellular metabolism.

It has also been shown that signaling pathways such as the transforming growth factor β (TGF-β)/serine-threonine kinase (Ser/Thr kinase) receptor complex are also involved in the phenotypic transformation of hypoxia-induced PASMCs. Hypoxia activates this signaling pathways, leading to a change in PASMC phenotype from contractile to synthetic, which can be attenuated by the corresponding inhibitors, demonstrating that this pathway is involved in the process of hypoxia-induced phenotypic conversion of PASMCs [15,16,17]. Meanwhile, the inhibition of PDK1 delayed lactic acid production, cell adhesion, migration, invasion, and angiogenesis—and, thus, metastasis—whereas overexpression of PDK1 showed the opposite effect [18]. Administration of the selective PDK inhibitor dichloroacetic acid (DCA) to experimental rats was able to reverse pulmonary arterial hypertension, suggesting that PDK has an important role in the process of pulmonary arterial hypertension [19,20]. In summary, although some studies have elucidated the important role of smooth muscle cells’ phenotypic transformation in vascular remodeling—and that overexpression, knockdown, disruption, or inhibition of PDK1 affects its physiological regulation of cells’ transcriptional regulation, cell migration, cell growth, differentiation, proliferation, and apoptosis—the specific mechanisms need to be further investigated.

Therefore, in the present study, we constructed stably transfected cell lines overexpressing PDK1 via lentiviral transfection of yak PASMCs, and we cultured yellow cattle and yak PASMCs, as well as PDK1-OEcon/OE, under normoxia and 10% O_2_ to explore the effects of hypoxia on the PDK1 and TGF-β/Smad signaling pathways in yak PASMCs, and to provide basic information to further elucidate the mechanism of the PDK1-mediated TGF-β/Smad signaling pathway in yak PASMCs.

## 2. Materials and Methods

### 2.1. Animal Ethics

The test animals were collected from a healthy newborn yellow cattle (Qinchuan cattle), and yak lung tissues (regardless of sex) were collected from slaughterhouses in Cooperative City, Gansu Province (altitude: ~3500 m) and Xi’an City, Shaanxi Province (altitude: ~1000 m) and placed in saline solution containing double antibiotics. All animal experiments were approved by the Committee for Ethical Use of Animals of the School of Life Sciences and Engineering, Northwest University for Nationalities. The collected samples were subjected to serum biochemical analysis and physical examination, and both the yellow cattles and yaks were healthy.

### 2.2. Cell Isolation and Culture

The surfaces of neonatal yellow cattle and yak lung tissues were completely immersed in double-antibiotic-containing saline preheated at 37 °C, and then the pulmonary arteries were separated and their lumens were opened with sterile surgical scissors and surgical forceps, washed with saline, and then sheared into small pieces of ~1 mm^3^.

Tissue adhesion method: Tissue blocks were adhered to the bottom surface of the culture flasks, and after they were tightly adhered, the flasks were injected with an appropriate amount of 15% fetal bovine serum (FBS) DME/F12 medium (Cellmax, Minhai Biological Engineering Co., Lanzhou, China) and slowly turned over and laid flat at 180° so that the blocks could be fully infiltrated and cultured. Trypsin digestion: Several tissue blocks were placed in a shake flask containing grinding beads, preheated 0.25% trypsin (Cellmax, Minhai Biological Engineering Co., Lanzhou, China) was added to completely submerge the tissue blocks, and the digestion was carried out at 37 °C for 20–40 min, with the mixture being shaken every 5 min. After the digestion was terminated, the cell suspension was allowed to stand for 5 min, and the filtered cell suspension was transferred to a centrifuge tube and centrifuged at 1000 rpm/min for 10 min, after which the supernatant was discarded. The cell density was adjusted to ~5 × 10^5^/mL and then transferred to T25 cell culture flasks for further cultivation. After some cells crawled out or adhered to the wall, the medium was changed regularly and the culture continued.

### 2.3. Cell Purification

Cells grown to 80–90% confluence, as described above, were completely covered with 0.25% trypsin and placed in the incubator for digestion, and the culture flasks were observed under an inverted microscope after 30 s. The smooth muscle cells were rounded and partially detached, and the medium was immediately added to complete the digestion. The growth area previously labeled by smooth muscle cells was gently blown under the microscope, avoiding the growth area of endothelial cells. After blowing, the medium containing pulmonary artery smooth muscle cells was transferred to another cell bottle for culture. Repeat passaging was performed for the above operation to differentiate endothelial cells from smooth muscle cells. After the cells had grown to 80–90%, they were digested with trypsin (30–60 s), the tissue mass was discarded, and some of the cells were frozen with cell cryopreservation solution (15% FBS DME/F12 medium:FBS:DMSO = 5:4:1).

### 2.4. Immunofluorescence

Sterile crawlers were placed in 12-well plates, and cell crawling was performed at 1~5 × 10^5^ cells/mL per well. First, when the cells grew to 80~90%, the medium was discarded and washed 3 times with PBS, and then 4% paraformaldehyde was added to each well to fix the crawler (1 h). Next, 100 μL of 0.1% Triton-X-100 was permeabilized into each well at room temperature (30 min) and washed with PBS 3 times (3 min/time), and then 100 μL of 5% BSA was added dropwise into each well to confine the crawler at room temperature (30 min). Then, 100 μL of primary antibody (dilution ratio of 1:100) was added dropwise into each well, and the crawler was incubated at 4 °C for 4 h, after which 100 μL of primary antibody (dilution 1:100) was added to each well, incubated for 12 h at 4 °C, and washed three times with PBS (3 min/time). Then, 100 μL of secondary antibody (dilution 1:50) was added to each well, incubated at 37 °C with light protection for 1 h, and washed 3 times (3 min/time) with PBS. Finally, DIPA-containing anti-fluorescence quenching sealer was added to the slide under light protection, and the slide was inverted and placed in a wet box. The slides were inverted and placed in a wet box for subsequent immunofluorescence identification of cells.

### 2.5. Cell Processing and Grouping

Using a CO_2_ cell culture incubator (BB15, Thermo Fisher Scientific Inc., Shanghai, China) and a three-gas incubator (N_2_, CO_2_, O_2_, CCL-050T-8, Esco Lifesciences Group, Shanghai, China) with a CO_2_ concentration of 5%, the above isolated and purified primary PASMCs from yellow cattle and and yaks were cultured under normoxia (20% O_2_ concentration, control group) and hypoxia (10% O_2_ concentration, hypoxia 10 group) at a constant temperature of 37 °C.

### 2.6. Construction of a Stable Transient Cell Line of PDK1-Overexpressing Yak PASMCs

#### 2.6.1. Construction of PDK1 Overexpression Lentiviral Vector

The fragment of the PDK1 target gene was prepared via PCR amplification, and the linearized vector (GV492) was digested by restriction endonuclease. The linearized vector and the target gene amplification product were used to formulate the reaction system, the recombinant product was obtained for direct transformation, the single clones on the plate were picked for PCR identification, and the positive clones were subjected to sequencing and result analysis. The correct clone was expanded and cultured, extracted, and the tool vector plasmid carrying the target gene PDK1 with high purity was obtained. The sequence of vector elements was Ubi-MCS-3FLAG-CBh-gcGFP-IRES-puromycin, its plasmid was co-transfected with the viral packaging auxiliary plasmids Helper 1.0 and 2.0 into 293T cells, and the virus harvest was performed at 48~72 h after the completion of transfection. The virus was harvested at 72 h after transfection, the high-titer lentiviral maintenance solution was obtained after concentration and purification, the overexpression of PDK1 lentivirus was obtained after passing the quality inspection, and the lentiviral blank vector control was obtained as described above.

#### 2.6.2. Lentiviral Transfection and Screening

Yak PASMCs in good growth condition were plated in 24-well plates at 4 × 10^4^/well, and DME/F12 complete medium with 10% FBS was added at 1 mL/well. When the cells grew to approximately 60%, one well was randomly selected for cell counting. In the biosafety cabinet, the transfection solution was prepared as shown in Table 1, and the lentiviral transfection solution was added to 24-well plates at 500 μL per well. After 12 h of culture, the transfection solution was discarded, washed three times with PBS, and replaced with new complete medium, and the culture was expanded after the cells in the wells had grown to ~90% and used for freezing and subsequent experiments. The MOI values were supported by laboratory data from the previous experiments, and the optimal MOIs for overexpression of PDK1 and its blank vector control lentivirus were determined to be 50.

The transiently transfected PDK1-overexpressing yak PASMCs, and its blank vector control lentiviral yak PASMCs, as described above, with cells in good condition after passaging, were screened using complete medium containing 4 μg/mL puromycin; after 3 generations of screening, expression was verified, and after passaging, the stably transfected PDK1-overexpressing cells (PDK1-OE) and their blank vector control (PDK1-OEcon) were confirmed in the yak PASMC strains. The puromycin screening concentration was supported by the prelaboratory data, determined to be 4 μg/mL for the overexpression of PDK1 and its blank vector control lentivirus.

### 2.7. Cell Growth Curve

The cell suspensions were prepared by digesting the well-grown yellow cattle and yak wall-adherent PASMCs (using generations 4–6) and stable OEcon/OE yak PASMCs (using generation 3), and then spreading them in 24-well plates at a cell density of 1 × 10^4^/mL (1 mL per well) and spreading the cell suspensions evenly on the bottom of the plates using the “8” shaking method; after the cells adhered to the wall, they were cultured and collected at different timepoints (day 1, 2, 3, etc.), cleaned, digested, and then prepared as cell suspensions, and a small amount of suspension was added to the cell counting plate and placed in the cell counter for counting. Three wells were repeated for each sample, and the average value was calculated to plot the growth curve.

The cell multiplication time was calculated according to the growth curve, by taking the cell count (Y), inoculated cell count (X), and growth time (T) of the day before the cell peak. The formula is as follows:

Multiplication time = T/A       A = log_2_ (Y/X).

### 2.8. Apoptosis Analysis

The cells were washed by means of centrifugation with precooled PBS, and 1–10 × 10^5^ yellow cattle and yak PASMCs and stably transformed OEcon/OE yak PASMCs (including cells in culture supernatant) were collected. We diluted 5× Binding Buffer with double-distilled water to 1× working solution, and then we took 500 μL of 1× Binding Buffer to resuspend the cells. Then, we added 5 μL of Annexin V-APC and 10 μL of 7-AAD to each tube, gently vortexed them to mix them well, and incubated them at room temperature away from light for 5 min, before detection via flow cytometry. Each sample was repeated three times.

### 2.9. ROS Analysis

The cells were washed by means of centrifugation with precooled PBS, and 1–10 × 10^5^ yellow cattle and yak PASMCs and stably transformed OEcon/OE yak PASMCs were collected. Serum-free medium was used to dilute the DHE probe 1:1000, 100 μL of cell suspension was aspirated into a flow-through sample tube, and 300 μL of formulated DHE probe was added and incubated for 30 min in the dark. Then, 5 mL of PBS was added to mix well and then centrifuged at 300× *g* for 5 min to wash away the unbound probe, after which 300 μL of PBS was added to each tube to resuspend the cell precipitate and detected via flow cytometry. Each sample was repeated three times.

### 2.10. Cell Scratching Analysis

After digestion of well-grown yellow cattle and yak PASMCs (using generations 4–6) and stabilized OEcon/OE yak PASMCs (using generation 3), the medium was terminally digested, and the cell suspension was prepared by means of blowing and mixing before being spread in 6-well plates at a cell density of 3~5 × 10^5^/mL (1 mL per well) and supplemented with 1 mL of complete medium, and then spread evenly on the bottom of the plate using the “8” shaking method. After the cells had adhered to the wall and grown to approximately 90%, the center of the cells was gently scratched along a straight line with a 200 μL tip, washed three times with PBS, and then serum-free DME/F12 medium was added, after which each group of cells was cultured every 6 h. New serum-free medium was added for observation and photography. Each sample was repeated for 3 wells. The area of the images was measured using ImageJ to calculate the migration rate of the cells.

### 2.11. Glucose and Lactic Acid Analysis

A total of 8.8 × 10^5^ yellow cattle and yak PASMCs (using generations 4–6) and stabilized OEcon/OE yak PASMCs (using generation 3) were inoculated in T25 cell flasks, and 10% FBS DME/F12 medium was added up to 8 mL. The medium was collected from different groups of cells after culturing them for 24 h, 48 h, and 72 h; 20 μL of the calibration solution was added to the biosensor analyzer for calibration. After calibration, 20 μL of the sample diluted 3–5 times with pure water was added to the analyzer to determine glucose and lactate concentrations. Each sample was repeated three times, and the average was calculated.

### 2.12. RT-qPCR Analysis

RNA was extracted from yellow cattle, yak, and stably transformed OEcon/OE-yak PASMCs cultured for 24, 48, and 72 h in the control and hypoxia groups, and cDNA was obtained after reverse transcription and stored at −20 °C for subsequent experiments. The total system of the RT-qPCR reaction was 20 μL, including 8.2 μL of H_2_O, 0.8 μL of the abovementioned synthesized primers, 10 μL of 2× Universal SYBR Green Fast qPCR Mix, and 1 μL of cDNA; the sequence of the primers is shown in Table S1. The reaction conditions were as follows: pre-denaturation at 95 °C for 30 s, denaturation at 95 °C for 5 s, and annealing at 60 °C for 30 s, for a total of 40 cycles, followed by final annealing. RT-qPCR reactions were performed in a fluorescence quantitative PCR instrument (CFX96, Bio-Rad Laboratories, Hercules, CA, USA), and the values obtained were normalized to the internal reference β-actin. The RT-qPCR results were analyzed using the 2^−ΔΔCt^ method.

### 2.13. Western Blot Analysis

The samples of stably transformed OEcon/OE yak PASMCs cultured in the control group were collected, the protein concentration was determined via the BCA method, and the protein concentration of each sample was adjusted to 1.5 μg/μL to obtain the protein samples. According to the molecular weight of the protein run, different concentrations of separator and concentrate gels were configured, the prepared protein samples were spotted into the gel wells with a marker upsampling volume of 5 μL, and SDS-PAGE gel electrophoresis was followed by membrane transfer using TransBlot (Bio-Rad, Bio-Rad Laboratories, Hercules, CA, USA). The protein bands were transferred to a PVDF membrane under the following conditions: 13~15 V, 0.5–1 A, 20 min; after completion, the membrane was blocked with TBST solution containing 5% skimmed milk for 2 h at room temperature; the primary antibody (1:1000 PDK1; 1:1000 β-actin) was added and incubated at 4 °C overnight, and the membrane was washed with TBST three times (5 min/times); the corresponding secondary antibody was diluted with the blocking solution, incubated for 50 min at room temperature, and then washed with TBST three times (5 min/time). ECL (enhanced chemiluminescence) solution was added dropwise and placed in the chemiluminescence instrument for color development. The resulting protein bands were analyzed for gray scale values as shown in Image J (1.8.0_172).

### 2.14. Statistics and Analysis

All measurement data are expressed as the mean ± standard error (mean ± SEM), and data were analyzed using GraphPad Prism 9 software for conformity to normal distribution and chi-squared. Comparative analysis was performed using one-way or two-way ANOVA, with significance determined as follows: *p* < 0.05, significant; *p* < 0.001, highly significant; *p* > 0.05, not significant.

## 3. Results

### 3.1. Yellow Cattle and Yak Primary PASMC Isolation, Culture Purification, and Characterization

**Figure 1:**
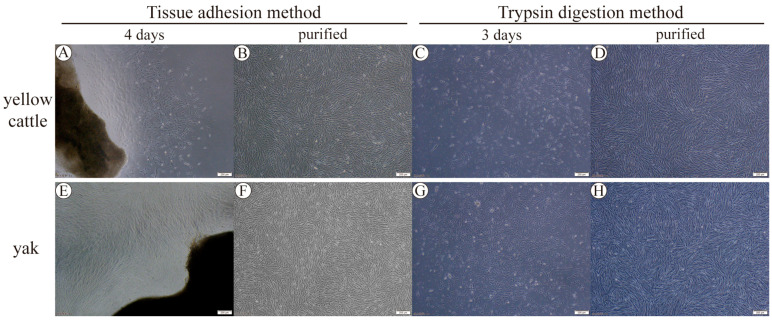
Isolation and culture purification of PASMCs via tissue adhesion and trypsin digestion methods: (**A**,**B**) PASMCs isolated, cultured, and purified from yellow cattle pulmonary arteries via the tissue adhesion method; (**C**,**D**) PASMCs isolated, cultured, and purified from yellow cattle pulmonary arteries via the trypsin digestion method; (**E**,**F**) PASMCs isolated, cultured, and purified from yak pulmonary arteries via the tissue adhesion method; (**G**,**H**) PASMCs isolated, cultured, and purified from yellow cattle pulmonary arteries via the trypsin digestion method; 40×.

**Figure 2:**
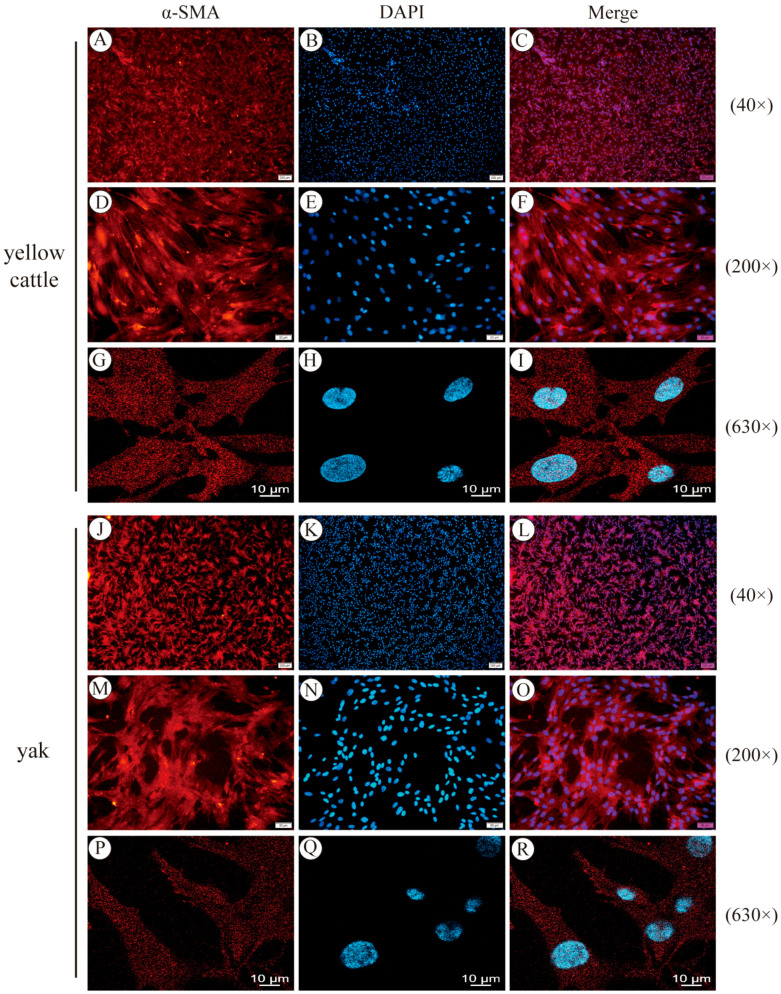
Immunofluorescence identification and detection of α-SMA expression in PASMCs: (**A**–**C**) results of immunofluorescence staining of yellow cattle PASMCs at 40×; (**D**–**F**) results of immunofluorescence staining of yellow cattle PASMCs at 200×; (**G**–**I**) results of immunofluorescence staining of yellow cattle PASMCs at 630×; (**J**–**L**) results of immunofluorescence staining of yak PASMCs at 40×; (**M**–**O**) results of immunofluorescence staining of yak PASMCs at 200×; (**P**–**R**) results of immunofluorescence staining of yak PASMCs at 630×.

It was found that the cells at the bottom of the culture flasks were in the shape of long pikes, irregular, triangular, or fan-shaped, which first proved the isolation and cultivation of yellow cattle and yak PASMCs (Figure 1A,C,E,G). Then, by using the different tolerance of smooth muscle cells and endothelial cells to trypsin, the smooth muscle cells were digested down before the endothelial cells to separate the two, purifying the cells. Observed under the microscope (Figure 1B,D,F,H), the cells after digestion, separation, and purification had the morphological characteristics of smooth muscle cells and possessed the characteristics of growth. At low cell densities, they were often intertwined in the form of a net, and at high densities, when the cell density was low, the cells were often intertwined into a mesh, and when the density was high, the cells were arranged in a vortex or fenestrated shape; the cell growth fusion reached 80% at around 3 d after the primary yellow cattle and yak PASMCs, which was a better growth state. α-SMA is a characteristic molecule of SMCs; the cultured generation-3 PASMCs were stained for immunofluorescence, and the inverted microscope and confocal laser microscope were used to observe that the α-SMA was arranged in myofilaments in the cytoplasm, the cytoplasm was stained red, and the nucleus were stained blue. The positive cell rate was more than 95% (Figure 2), indicating that the cultured primary cells were of high purity. The above results indicate that the yellow cattle and yak PASMCs were successfully isolated and purified, meeting the needs of subsequent experiments.

### 3.2. Construction of a Stable Transient Cell Line of PDK1-Overexpressing Yak PASMCs

**Figure 3:**
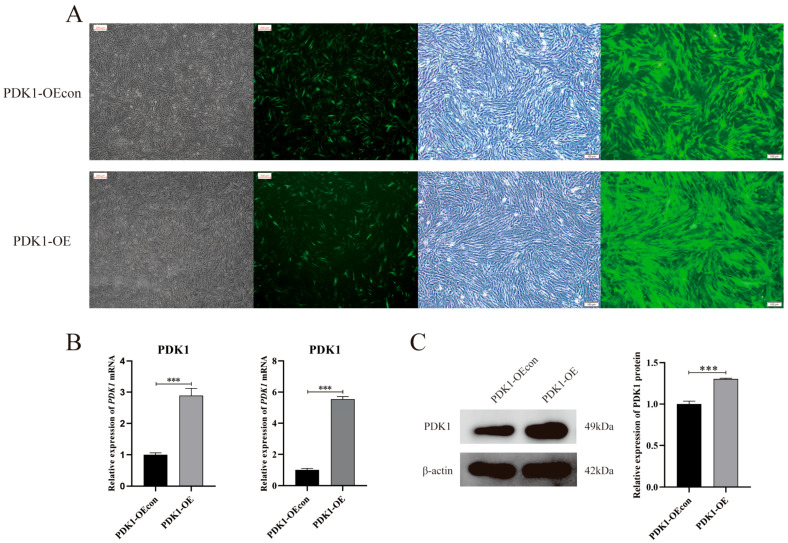
Construction and validation of stably transfected cell lines overexpressing PDK1 yak PASMCs: (**A**) bright field and fluorescence images of transiently transfected and stably transfected yak PASMCs of PDK1-OEcon and PDK1-OE, 100×; (**B**) relative expression of PDK1 mRNA in transiently transfected and stably transfected yak PASMCs of PDK1-OEcon and PDK1-OE; (**C**) PDK1-OEcon and PDK1-OE PDK1 protein bands and relative expression of stably transfected yak PASMCs; *** indicates a highly significant difference (*p* < 0.001).

After lentiviral transfection, the fluorescence results of PDK1-overexpressing (PDK1-OE group) and its blank vector control (PDK1-OEcon group) yak PASMCs were found to be transiently transfected cells were better transfected, and the fluorescence of the two was detected after three generations of puromycin screening, with a high fluorescence intensity and a positive cell rate of more than 90% (Figure 3A). The mRNA and protein expression of PDK1 were also detected by means of RT-qPCR and Western blotting, and it was found that the relative expression of PDK1 was significantly increased (*p* < 0.001) (Figure 3B,C). The successful construction of stable transient cell lines overexpressing PDK1 and its blank vector control yak PASMCs was demonstrated.

### 3.3. Effect of Hypoxia on Proliferation Levels of Yellow Cattle, Yak, and Their PDK1-Overexpressing PASMCs

**Figure 4:**
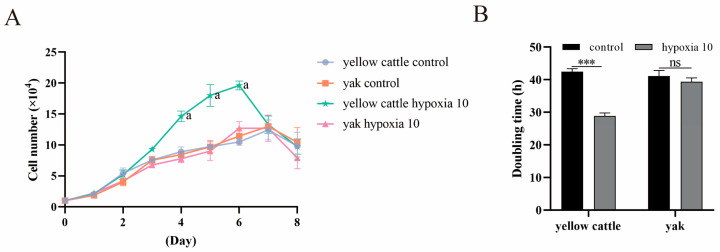
Proliferation level assay of yellow cattle and yak PASMCs: (**A**) growth curves of yellow cattle and yak PASMCs under normoxic and hypoxic conditions; the graph compares different subgroups at the same time period; comparisons between groups labeled with a letter are significantly different (*p* < 0.05), and comparisons between groups without a letter are not significantly different (*p* > 0.05). (**B**) Comparison of multiplication time of yellow cattle and yak PASMCs under normoxic and hypoxic conditions; ns indicates the difference is not significant (*p* > 0.05), *** indicates a highly significant difference (*p* < 0.001).

**Figure 5:**
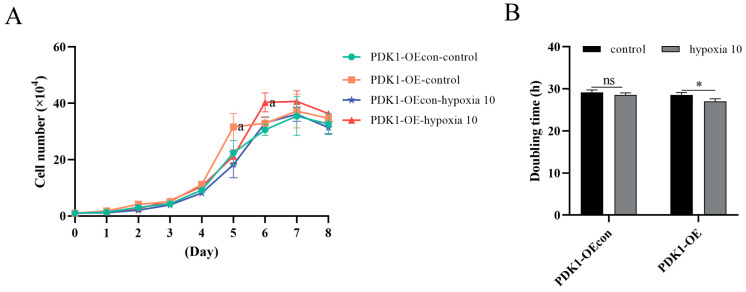
Proliferation level assay of yak PASMCs overexpressing PDK1: (**A**) Growth curves of yak PASMCs under normoxic and hypoxic conditions with PDK1 overexpression; comparisons between groups labeled with a letter are significantly different (*p* < 0.05), and comparisons between groups without a letter are not significantly different (*p* > 0.05). (**B**) comparison of multiplication times of yak PASMCs under normoxic and hypoxic conditions with PDK1 overexpression; ns indicates the difference is not significant (*p* > 0.05), * indicates the difference is significant (*p* < 0.05).

It was found that under hypoxia, the multiplication time of yellow cattle PASMCs was shortened from ~42 h to ~28 h compared to normoxia, and the proliferation level was significantly increased (*p* < 0.001); the effect of hypoxia on the proliferation of yak PASMCs was not significant (*p* > 0.05) (Figure 4). Under the condition of hypoxia, the proliferation time of PDK1-OE yak PASMCs was shortened from ~28.5 h to ~27 h compared to that under normoxia, and the proliferation level was significantly increased (*p* < 0.05); the effect of hypoxia on the proliferation of PDK1-OEcon yak PASMCs was not significant (*p* > 0.05) (Figure 5). This indicates that hypoxia promotes the proliferation of PASMCs in yellow cattles and has no effect on the proliferation of PASMCs in yaks; overexpression of PDK1 promotes the proliferation of PASMCs in yaks, and hypoxia promotes the overexpression of PDK1 in the proliferation of PASMCs in yaks.

### 3.4. Effect of Hypoxia on Apoptosis Levels in Yellow Cattle, Yak, and Their PDK1-Overexpressing PASMCs

**Figure 6:**
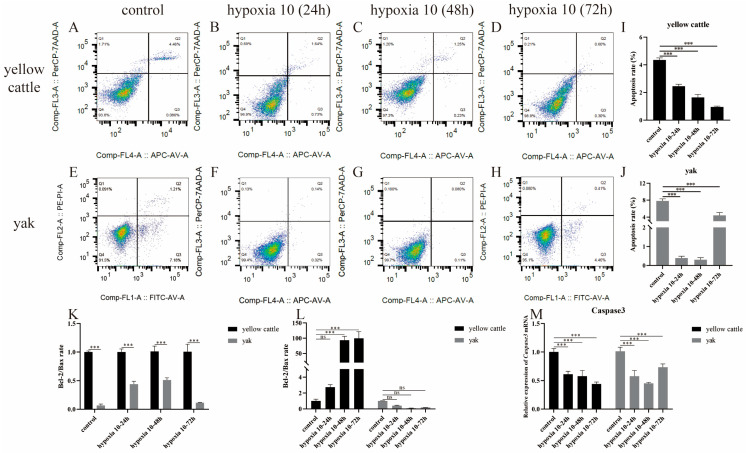
Detection of apoptosis levels of yellow cattle and yak PASMCs: (**A**–**D**) distribution of apoptosis in yellow cattle PASMCs under normoxic and varying-duration hypoxic conditions; (**E**–**H**) distribution of apoptosis in yak PASMCs under normoxic and varying-duration hypoxic conditions; (**I**) apoptosis rate of yellow cattle PASMCs under normoxic and varying-duration hypoxic conditions; (**J**) apoptosis rate of yak PASMCs under normoxic and varying-duration hypoxic conditions; (**K**,**L**) levels of Bcl-2/Bax anti-apoptotic ratio in yellow cattle and yak PASMCs; (**M**) relative mRNA expression of the apoptotic gene Caspase3 in yellow cattle and yak PASMCs under normoxic and varying-duration hypoxic conditions. ns indicates the difference is not significant (*p* > 0.05), *** indicates a highly significant difference (*p* < 0.001).

**Figure 7:**
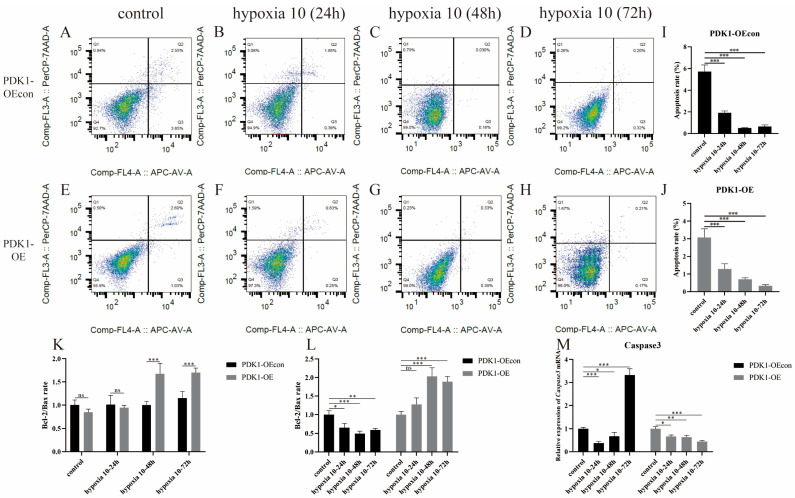
Detection of apoptosis levels in yak PASMCs overexpressing PDK1: (**A**–**D**) apoptosis distribution maps of PDK1-OEcon under normoxic and varying-duration hypoxic conditions; (**E**–**H**) apoptosis distribution maps of PDK1-OE under normoxic and varying-duration hypoxic conditions; (**I**) apoptosis rate of PDK1-OEcon under normoxic and varying-duration hypoxic conditions; (**J**) apoptosis rate of PDK1-OE under normoxic and varying-duration hypoxic conditions; (**K**,**L**) results of the detection of the level of anti-apoptosis Bcl-2/Bax ratio in yak PASMCs overexpressing PDK1; (**M**) relative mRNA expression of the apoptotic gene Caspase3 in yak PASMCs overexpressing PDK1 under normoxic and varying-duration hypoxic conditions; ns indicates the difference is not significant (*p* > 0.05), * indicates the difference is significant (*p* < 0.05), ** indicates the difference is significant (*p* < 0.01), *** indicates a highly significant difference (*p* < 0.001).

With the change in incubation time under hypoxic conditions, the apoptosis level of yellow cattle PASMCs showed a decreasing trend by flow cytometry (Figure 6A,B,I), and the apoptosis level of yak PASMCs showed a decreasing and then increasing trend (Figure 6E–H,J), which was consistent with the expression trend of anti-apoptosis indicator, B-cell lymphoma 2 (Bcl-2)/Bax ratio, and apoptosis gene, Caspase3 (Figure 6K–M). The apoptosis level of PASMCs from yaks stably transfected with PDK1-OEcon showed a decreasing and then an increasing trend (Figure 7A–D,I), which was consistent with the expression trend of the anti-apoptosis indicator Bcl-2/Bax ratio (Figure 7K,L), and the apoptosis level of PASMCs from yaks stably transfected with PDK1-OE showed a decreasing trend (Figure 7E–H,J), and the expression of anti-apoptosis indicator Bcl-2/Bax ratio showed a trend of first increase and then decrease (Figure 7K,L); meanwhile, the expression trend of apoptosis gene Caspase3 was consistent in both (Figure 7M). The steady transfection of PDK1-OE yak PASMCs and yellow cattle PASMCs (Figure 6) was similar in hypoxic apoptosis and anti-apoptosis levels. It indicates that with the change in hypoxia incubation time, hypoxia inhibited the apoptosis of yellow cattle PASMCs, and long-term hypoxia promoted the apoptosis of yak PASMCs; overexpression of PDK1 inhibited the apoptosis of yak PASMCs, and hypoxia promoted the inhibitory effect of overexpression of PDK1 on the apoptosis of yak PASMCs. It is suggested that the low level of anti-apoptosis and high level of apoptosis in short-term hypoxia in yellow cattle PASMCs may be related to the compensation of their short-term hypoxic stress cells.

### 3.5. Effect of Hypoxia on Migration Levels of Yellow Cattle, Yak and Their PDK1-Overexpressing PASMCs

**Figure 8:**
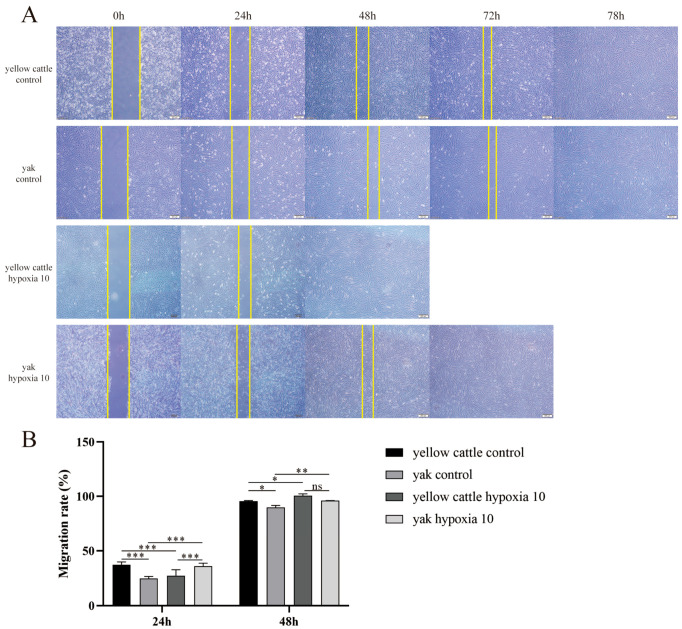
Migration level assay of yellow cattle and yak PASMCs: (**A**) scratch experiments of yellow cattle and yak PASMCs under normoxic and hypoxic conditions; (**B**) migration rates of yellow cattle and yak PASMCs under normoxic and hypoxic conditions at different times; ns indicates the difference is not significant (*p* > 0.05), * indicates the difference is significant (*p* < 0.05), ** indicates the difference is significant (*p* < 0.01), *** indicates a highly significant difference (*p* < 0.001).

**Figure 9:**
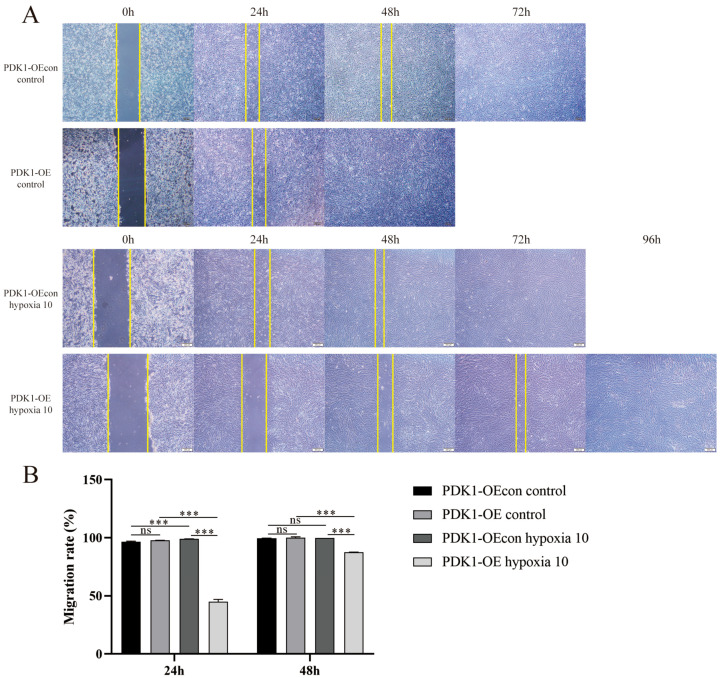
Migration level assay of yak PASMCs overexpressing PDK1: (**A**) scratch assay of yak PASMCs overexpressing PDK1 under normoxic and hypoxic conditions; (**B**) migration rates of yak PASMCs overexpressing PDK1 under normoxic and hypoxic conditions at different times; ns indicates the difference is not significant (*p* > 0.05), *** indicates a highly significant difference (*p* < 0.001).

The results obtained from the scratch experiment showed that there were no significant differences between the yellow cattle and yak PASMCs in the control group, both of which were completely healed at approximately 78 h (Figure 8); the migration level of PDK1-OE yak PASMCs was stronger than that of PDK1-OEcon cells, which were completely healed at approximately 48 h (Figure 9). Under hypoxic conditions, the migration ability of yellow cattle PASMCs was enhanced, and they were completely healed at ~48 h. Yak PASMCs had a significantly higher hypoxic migration rate than under normoxic conditions at 48 h (*p* < 0.05), and they were completely healed at ~72 h under hypoxic conditions, where the healing time was not significantly different from that under normoxic conditions (*p* > 0.05) (Figure 8). The migration of PDK1-OE yak PASMCs was significantly slowed (*p* < 0.001), and they were completely healed at 96 h. The healing time of PDK1-OEcon yak PASMCs under hypoxia was not significantly different from that under normoxia, and they were all completely healed at 72 h (Figure 9). This indicates that hypoxia promoted the migration of yellow cattle PASMCs and had no effect on the migration of yak PASMCs. PDK1 overexpression promoted the migration of yak PASMCs, and hypoxia inhibited the migration of yak PASMCs overexpressing PDK1.

### 3.6. Effects of Hypoxia on Glucose Metabolism Function in Yellow Cattle, Yak, and Their PDK1-Overexpressing PASMCs

**Figure 10:**
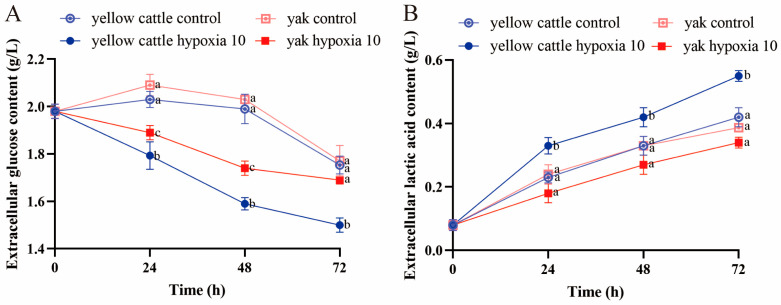
Glucose metabolism function assay of yellow cattle and yak PASMCs: (**A**) trend graphs of changes in extracellular glucose content under normoxic and hypoxic conditions at different times in yellow cattle and yak PASMCs; (**B**) trend graphs of changes in extracellular lactate content under normoxic and hypoxic conditions at different times in yellow cattle and yak PASMCs; lowercase letters a–c refer to the variability of comparisons between different groups; comparisons between groups labeled with a letter are significantly different (*p* < 0.05), and comparisons between groups without a letter are not significantly different (*p* > 0.05).

**Figure 11:**
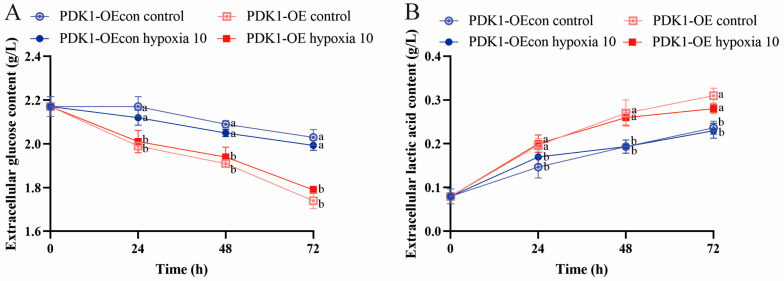
Detection of glucose metabolism function in yak PASMCs overexpressing PDK1: (**A**) trend graphs of changes in extracellular glucose content under normoxic and hypoxic conditions at different times with the overexpression of PDK1 on yak PASMCs; (**B**) trend graphs of changes in extracellular lactate content under normoxic and hypoxic conditions at different times with the overexpression of PDK1 on yak PASMCs; lowercase letters a,b refer to the variability of comparisons between different groups; comparisons between groups labeled with a letter are significantly different (*p* < 0.05), and comparisons between groups without a letter are not significantly different (*p* > 0.05).

By examining glucose and lactate in the cell culture medium of different groups, it was found that the trend of changes in the extracellular glucose contents in yellow cattle and yak PASMCs under normoxic conditions was consistent, both increasing and then decreasing, with no significant differences (*p* > 0.05); at 24 and 48 h of hypoxia, there was a significant difference between yellow cattle and yak PASMCs, but at 72 h of hypoxia, there was a significant difference between in the yellow cattle cells under normoxic conditions (*p* < 0.05), while there was no significant difference in the yak PASMCs (*p* > 0.05) (Figure 10A). The changes in the extracellular lactate content of yellow cattle PASMCs under hypoxic conditions were significantly greater than those under normoxic conditions (*p* < 0.05) and increased with the prolongation of the hypoxic incubation time, whereas the changes in the extracellular lactate content of yak PASMCs were not significantly different under either normoxic or hypoxic conditions (*p* > 0.05) (Figure 10B). The differences in the extracellular glucose and lactate contents of PDK1-OE yak PASMCs were significant (*p* < 0.05) compared with the control group under each time condition of hypoxia; however, the effect on PDK1-OE yak PASMCs’ glucose and lactate contents was not significant under different oxygen concentration conditions (*p* > 0.05) (Figure 11). Hypoxia promoted glucose consumption and lactate production in yellow cattle PASMCs, and long-term hypoxia had no significant effect on yak PASMCs; overexpression of PDK1 promoted glucose consumption and lactate production in yak PASMCs, and hypoxia had no effect on glucose consumption and lactate production in yak PASMCs overexpressing PDK1.

### 3.7. Effects of Hypoxia on ROS Levels in Yellow Cattle, Yak, and Their PDK1-Overexpressing PASMCs

**Figure 12:**
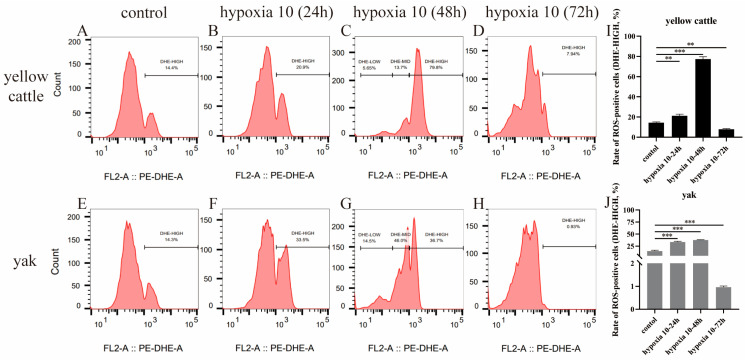
Detection of ROS levels within yellow cattle and yak PASMCs: (**A**–**D**) plot of ROS levels of PASMCs in yellow cattle under normoxic and varying-duration hypoxic conditions; (**E**–**H**) plot of ROS levels in yak PASMCs under normoxic and varying-duration hypoxic conditions; (**I**) comparison of changes in ROS levels in yellow cattle PASMCs under normoxic and varying-duration hypoxic conditions; (**J**) comparison of changes in ROS levels in yak PASMCs under normoxic and varying-duration hypoxic conditions; ** indicates the difference is significant (*p* < 0.01), *** indicates a highly significant difference (*p* < 0.001).

**Figure 13:**
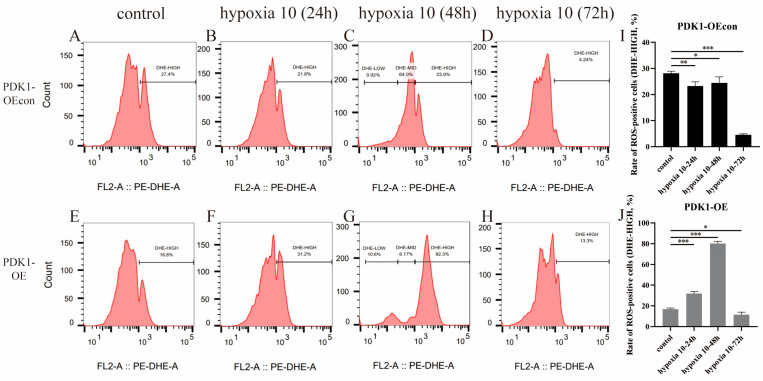
Detection of ROS levels within yak PASMCs overexpressing PDK1: (**A**–**D**) plot of ROS levels of PDK1-OEcon cells under normoxic and varying-duration hypoxic conditions; (**E**–**H**) plot of ROS levels of PDK1-OE cells under normoxic and varying-duration hypoxic conditions; (**I**) comparison of changes in ROS levels of PDK1-OEcon cells under normoxic and varying-duration hypoxic conditions; (**J**) comparison of changes in ROS levels in PDK1-OE cells under normoxic and varying-duration hypoxic conditions; * indicates the difference is significant (*p* < 0.05), ** indicates the difference is significant (*p* < 0.01), *** indicates a highly significant difference (*p* < 0.001).

With the change in incubation time under hypoxic conditions, flow cytometry showed that the ROS levels of both yellow cattle and yak PASMCs showed an increasing and then decreasing trend, as shown in Figure 12. Both had the highest ROS levels at 48 h of hypoxia, at 77.27% and 37.53%, respectively, while their lowest ROS levels were observed at 72 h of hypoxia, and the yak PASMCs (0.965%) had significantly lower ROS levels than the yellow cattle PASMCs (7.94%). The difference in ROS levels between the two was not significant under normoxia (*p* > 0.05). As shown in Figure 13, the ROS levels of PDK1-OEcon showed a decreasing trend, followed by an increasing trend, and then another decreasing trend, while those of PDK1-OE showed an increasing trend followed by a decreasing trend. pDK1-OEcon and PDK1-OE both had the highest ROS levels at 48 h of hypoxia, at 24.47% and 80.10%, respectively; both had their lowest ROS levels at 72 h of hypoxia, where that of PDK1-OE (11.65%) was significantly higher than that of PDK1-OEcon (4.58%). This indicates that hypoxia significantly increased the ROS levels in yellow cattle PASMCs and PDK1-overexpressing yak PASMCs compared with yak PASMCs.

### 3.8. Effects of Hypoxia on the Expression of Genes Related to the PDK1 and TGF-β/Smad Signaling Pathways in Yellow Cattle, Yak and Their PDK1-Overexpressing PASMCs

#### 3.8.1. Effect of Hypoxia on the Expression of PDK1 and TGF-β/Smad Pathway-Related Genes in Yellow Cattle and Yak PASMCs

**Figure 14:**
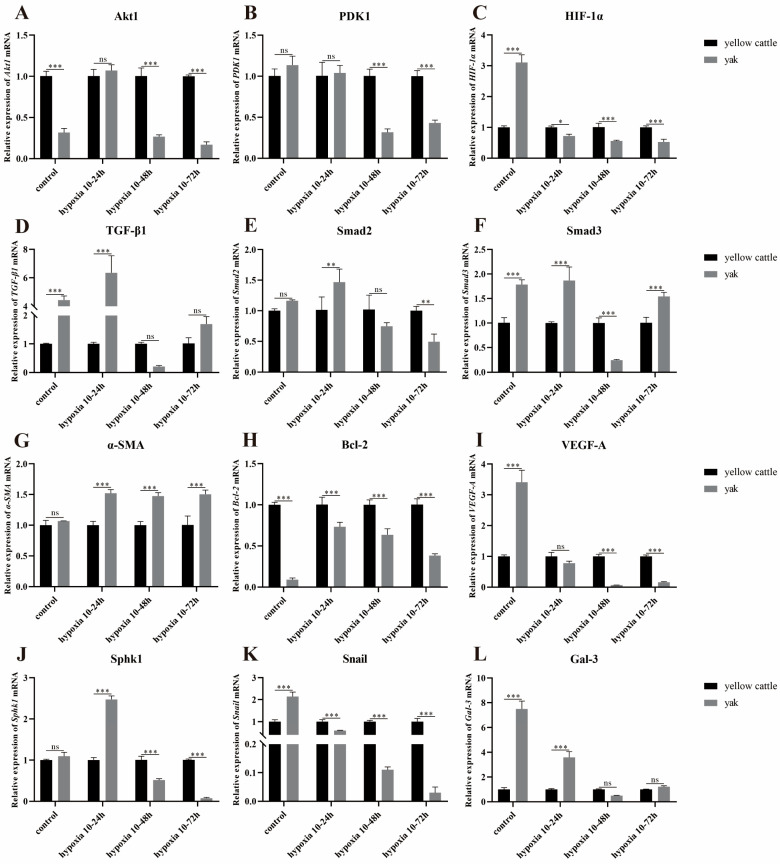
Expression of genes related to PDK1 and TGF-β/Smad signaling pathways in yellow cattle and yak PASMCs: (**A**–**L**) correspond to the relative mRNA expression of Akt1, PDK1, HIF-1α, TGF-β1, Smad2, Smad3, α-SMA, Bcl-2, VEGF-A, SphK1, Snail, and Gal-3 under normoxic and hypoxic conditions in yellow cattle and yak PASMCs, respectively. The results were normalized to the normoxic (control) group of yellow cattle; ns indicates the difference is not significant (*p* > 0.05), * indicates the difference is significant (*p* < 0.05), ** indicates the difference is significant (*p* < 0.01), *** indicates a highly significant difference (*p* < 0.001).

Under different time hypoxia groups, the relative expression of Akt1, PDK1, HIF-1α, Bcl-2, and Snail mRNA was lower in yak PASMCs compared with that in yellow cattle (Figure 14A–C,H,K); TGF-β1, Smad2, Smad3, α-SMA, SphK1, and Gal-3 mRNAs were all expressed in hypoxia 10–24 h with high relative expression (*p* < 0.001) (Figure 14D–G,J,L).

**Figure 15:**
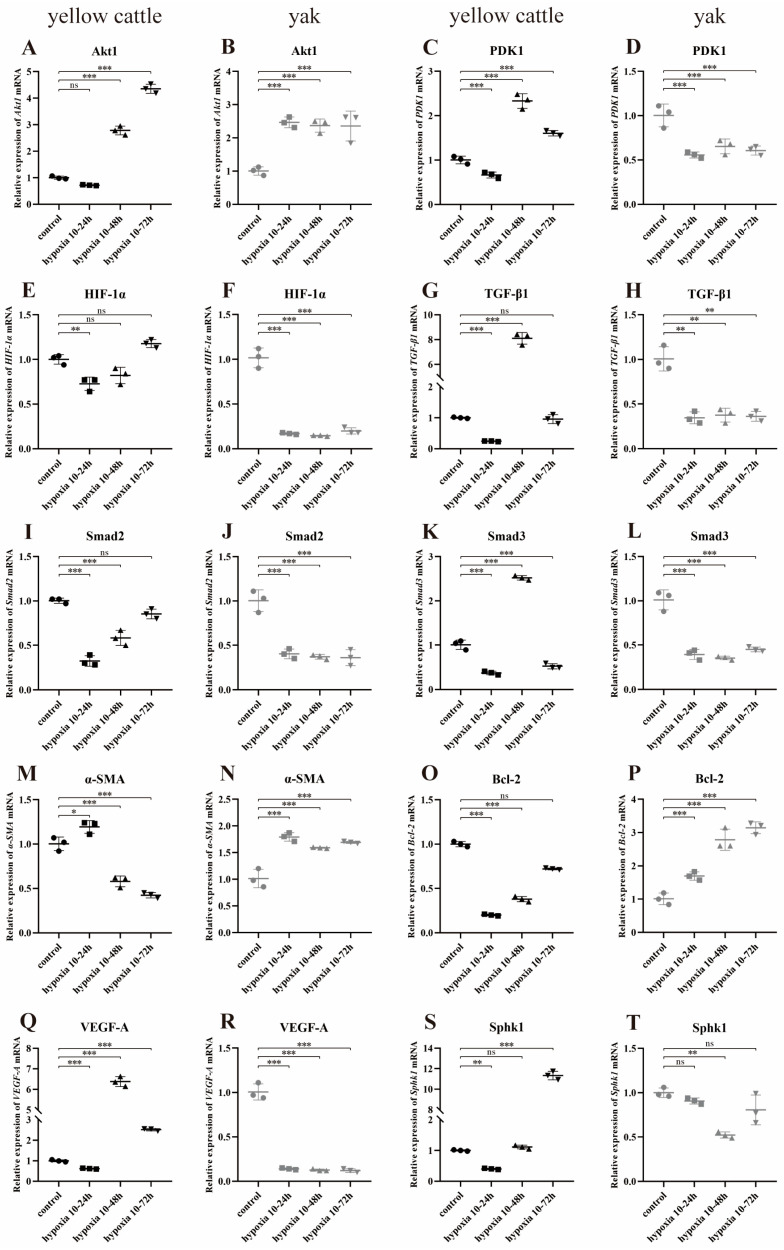

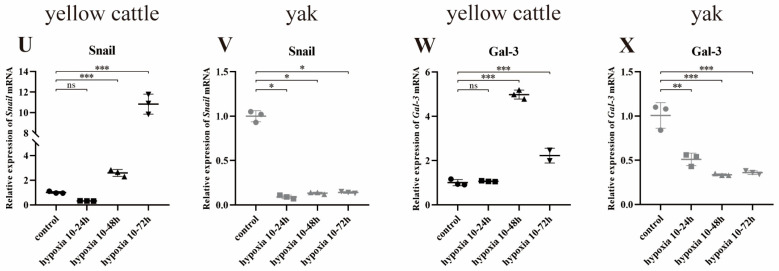
Expression of PDK1 and TGF-β/Smad pathway-related genes in yellow cattle and yak PASMCs under normoxic and various time-limited hypoxic conditions: (**A**,**C**,**E**,**G**,**I**,**K**,**M**,**O**,**Q**,**S**,**U**,**W**) are the relative mRNA expression of genes related to PDK1 and TGF-β/Smad signaling pathways in yellow cattle PASMCs; (**B**,**D**,**F**,**H**,**J**,**L**,**N**,**P**,**R**,**T**,**V**,**X**) are the relative mRNA expression of genes related to PDK1 and TGF-β/Smad signaling pathways in yak PASMCs. The results were normalized to the normoxic (control) groups of yellow cattle and yak, respectively; ns indicates the differ ence is not significant (*p* > 0.05), * indicates the difference is significant (*p* < 0.05), ** indicates the difference is significant (*p* < 0.01), *** indicates a highly significant difference (*p* < 0.001).

Meanwhile, with the change in cell hypoxia incubation time, the trends of Akt1, HIF-1α, Smad2, Bcl-2, SphK1, and Snail mRNA relative expression in yellow cattle PASMCs were consistent and increasing, and all of them had the highest expression at 10–72 h of hypoxia (Figure 15A,E,I,O,S,U); α-SMA was opposite and had the highest expression at 10–24 h of hypoxia (Figure 15M); the relative expression trends of PDK1, TGF-β1, Smad3, VEGF-A, and Gal-3 mRNAs were consistent with each other, showing an increasing and then decreasing trend, and all of them had the highest expression at 10–48 h of hypoxia (Figure 15C,G,K,Q,W). In yak PASMCs, except for Bcl-2 mRNA relative expression, which showed an increasing trend and was highest at 10–72 h of hypoxia (Figure 15P), and SphK1 mRNA relative expression, which showed a decreasing and then increasing trend and was lowest at 10–48 h of hypoxia (Figure 15T), the relative mRNA expression of the remaining genes did not change significantly (Figure 15B,D,F,H,J,L,N,R,V,X).

This suggests that compared with normoxia, hypoxia significantly affects the expression of genes related to the PDK1 and TGF-β/Smad signaling pathways in the PASMCs of yellow cattle and yak, which, in turn, promotes proliferation and angiogenesis and inhibits apoptosis; additionally, the cells were dedifferentiated from contractile to synthetic type, and inhibited angiogenesis in the PASMCs of yak, and the cells were contractile-type. Compared with yellow cattle, yak PASMCs adapted to the hypoxic environment by downregulating the expression of genes related to the PDK1 and TGF-β/Smad signaling pathways under hypoxic conditions.

#### 3.8.2. Effect of Hypoxia on the Expression of Genes Related to PDK1 and TGF-β/Smad Signaling Pathways in Yak PASMCs Overexpressing PDK1

**Figure 16:**
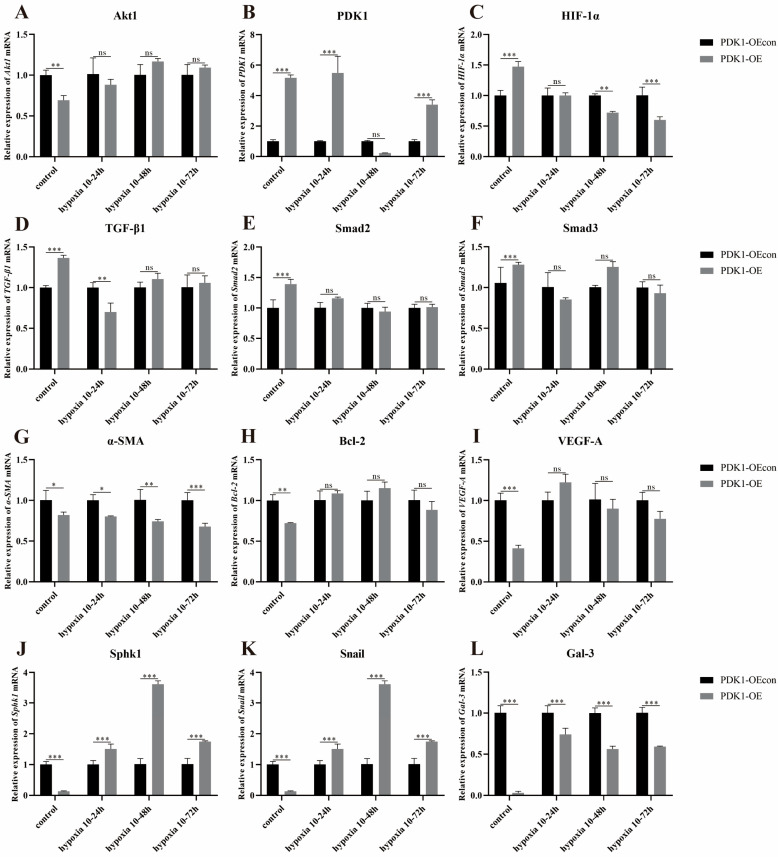
Overexpression of PDK1 on the expression of PDK1 and TGF-β/Smad signaling pathway-related genes in yak PASMCs under hypoxia: (**A**–**L**) Correspond to the relative mRNA expression of Akt1, PDK1, HIF-1α, TGF-β1, Smad2, Smad3, α-SMA, Bcl-2, VEGF-A, SphK1, Snail, and Gal-3 by overexpression of PDK1 in yak PASMCs under normoxic and hypoxic conditions, respectively. The results were normalized to the normoxia (control) group of PDK1-OEcon. ns indicates the difference is not significant (*p* > 0.05), * indicates the difference is significant (*p* < 0.05), ** indicates the difference is significant (*p* < 0.01), *** indicates a highly significant difference (*p* < 0.001).

Under different time hypoxia groups, the relative expression of PDK1, SphK1, and Snail mRNA was high in the PDK1-OE group compared with the PDK1-OEcon group (Figure 16B,J,K); the relative expression of HIF-1α, TGF-β1, and α-SMA mRNA was low at some time points (Figure 16C,D,G), and the relative expression differences in the remaining gene mRNAs were not significant (*p* > 0.05) (Figure 16A,E,F,H,I,L).

**Figure 17:**
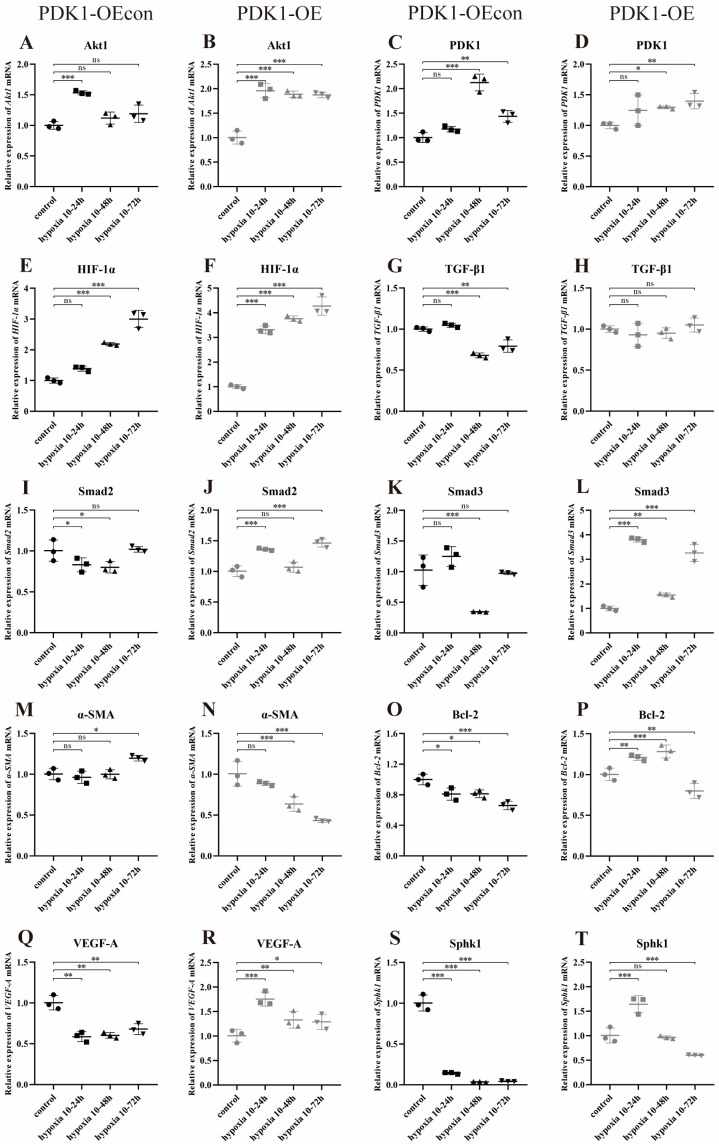

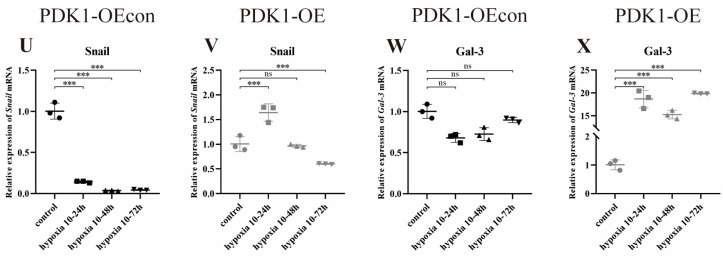
Expression of PDK1 and TGF-β/Smad pathway-related genes in PDK1-OEcon and PDK1-OE yak PASMCs under normoxic and various time-limited hypoxic conditions: (**A**,**C**,**E**,**G**,**I**,**K**,**M**,**O**,**Q**,**S**,**U**,**W**) are the relative mRNA expression of genes related to PDK1 and TGF-β/Smad signaling pathways in yellow cattle PASMCs; (**B**,**D**,**F**,**H**,**J**,**L**,**N**,**P**,**R**,**T**,**V**,**X**) are the relative mRNA expression of genes related to PDK1 and TGF-β/Smad signaling pathways in yak PASMCs. The results were normalized to the normoxic (control) groups of PDK1-OEcon and PDK1-OE, respectively; ns indicates the difference is not significant (*p* > 0.05), * indicates the difference is significant (*p* < 0.05), ** indicates the difference is significant (*p* < 0.01), *** indicates a highly significant difference (*p* < 0.001).

Meanwhile, with the change in hypoxia time, the relative expression of Akt1, TGF-β1, and Smad2/3 mRNA in PDK1-OEcon had a consistent trend of decreasing and then increasing, and all had the highest expression at hypoxia 10–24 h (Figure 17A,G,I,K); the relative expression of PDK1 mRNA had a trend of increasing and then decreasing, and had the highest expression at hypoxia 10–48 h (Figure 17C); the relative expression of HIF-1α, α-SMA, VEGF-A, and Gal-3 mRNA showed an increasing trend, and HIF-1α and α-SMA mRNA had the highest expression at hypoxia 10–72 h, and the expression of VEGF-A and Gal-3 mRNA was lower in hypoxia than the control group in each different time period (Figure 17E); the control group (Figure 17E,M,Q,W); the relative expression of Bcl-2, SphK1, and Snail mRNA showed a consistent trend of decreasing, all with the highest expression at hypoxia 10–24 h and lower than that of the control group (Figure 17O,S,U). The relative expression of PDK1 and HIF-1α mRNA in the PDK1-OE group showed an increasing trend, with the highest expression at hypoxia 10–72 h (Figure 17D,F); the relative expression of Smad2/3 and Gal-3 mRNA showed a decreasing and then increasing trend, both with high expression at hypoxia 10–24 h and 72 h (Figure 17J,L,X); the relative expression of α-SMA, VEGF-A, SphK1, and Snail mRNA showed a decreasing trend in relative expression, with the lowest expression at hypoxia 10–72 h (Figure 17N,R,T,V); Bcl-2 mRNA showed an increasing and then decreasing trend in relative expression, with the highest expression at hypoxia 10–48 h (Figure 17P). There was no significant change in the relative expression of the other genes (Figure 17B,H).

This suggests that compared with normoxia, hypoxia significantly affects the expression of genes related to the PDK1 and TGF-β/Smad signaling pathways in overexpressing PDK1 yak PASMCs, which, in turn, promotes proliferation and angiogenesis, inhibits apoptosis, and the cells were de-differentiated from contractile to synthetic phenotype in yak PASMCs.

## 4. Discussion

Humans or mammals from plains or low-elevation areas that live for a long time in the hypoxic environment of the plateau are prone to hypoxic pulmonary hypertension (HPH) and chronic respiratory diseases, where the occurrence of HPH is mainly related to pulmonary vascular remodeling. During pulmonary vascular remodeling induced by hypoxic conditions, endothelial cells, smooth muscle cells, fibroblasts, and even perivascular mesenchyme in the inner, middle, and outer layers of the vascular wall are involved in the process of vascular remodeling [21]. While PASMCs are the main components in the pulmonary vasculature, their abnormal proliferation, migration, and fibrosis of the inner and outer vascular membranes are the main causes of pulmonary vascular remodeling. Therefore, in this experiment, we investigated the phenotypic effects of yellow cattle and yak PASMCs under hypoxic concentrations and found that hypoxia could promote the proliferation, migration, and anti-apoptotic ability of yellow cattle PASMCs. Their proliferation time was shortened from ~42 h to ~28 h under normoxia; the scratches were completely healed at ~48 h under hypoxia; with the time of hypoxic culture, the ratio of the anti-apoptosis index Bcl-2/Bax gradually increased, and the anti-apoptosis level was lower at 24 h. Apoptosis was detected via flow cytometry, and the apoptosis gene Caspase3 was detected and showed a decreasing trend. In contrast, the proliferative capacity of yak PASMCs was not affected under hypoxia, and their level of apoptosis became increasingly high in the long term, while their scratch healing time was not significantly different from that under normoxia. The low level of anti-apoptosis in short-term hypoxic yellow cattle PASMCs may have been related to the compensatory adaptation of cells due to hypoxic stress in yellow cattles; after long-term hypoxic stimulation, yellow cattle PASMCs showed a similar phenotype to PASMCs in pulmonary vascular remodeling. It was tentatively concluded that prolonged hypoxia affects yellow cattle PASMCs and leads to pulmonary vascular remodeling. Meanwhile, under normal conditions, mature smooth muscle cells are highly differentiated into a contractile phenotype that facilitates muscle contraction and diastole, and smooth muscle cells dedifferentiated under external environmental stimuli represent a synthetic type of transformation. Synthetic smooth muscle with greater proliferative and migratory capacity will redifferentiate, which is one of the important reasons for the occurrence of irreversible vascular remodeling [22,23]. It was found that hypoxia induced a decrease in α-SMA, a marker of the contractile phenotype in PASMCs, along with an increase in the production of ROS and hydrogen peroxide in rats [24], whereas elevated levels of ROS oxidatively damage intracellular homeostasis, which, in turn, promotes the development of pulmonary hypertension. In this experiment, we found that the relative expression of α-SMA mRNA—a marker of a contractile cellular phenotype—and the levels of ROS were not significantly different between yellow cattle and yak PASMCs under normoxic conditions (*p* > 0.05). Under hypoxic conditions, the relative expression of α-SMA mRNA in short-term yellow cattle PASMCs increased, but it gradually decreased with the extension of incubation time, indicating that the yellow cattle PASMCs showed a contractile phenotype of dedifferentiation transformation; the relative expression of α-SMA mRNA in yak PASMCs under hypoxic conditions was significantly higher than that under normoxic conditions (*p* < 0.001), indicating that the yak PASMCs remained in a contractile phenotype. In terms of ROS levels, both yellow cattle and yak PASMCs showed a trend of increasing and then decreasing, but the ROS levels of yellow cattle PASMCs were higher than those of yak PASMCs in all hypoxic culture periods. In conclusion, these results show that hypoxia affects the phenotype and ROS levels of PASMCs in yellow cattles, leading to pulmonary vascular remodeling, which may cause the development of pulmonary hypertension; meanwhile, hypoxia has no significant effect on PASMCs in yaks, reflecting their good adaptability to hypoxia.

Hypoxia also affects the ability of intracellular glucose metabolism. It has been found that intracellular lactate synthesis increases under hypoxic conditions, and the accumulation of lactate activates the Raf-ERK signaling pathway, which promotes angiogenesis and cell growth [25]. The destination of pyruvate during gluconeogenesis is key to the cell’s selection of the type of glucose metabolism, which is mainly regulated by PDK [26]. The accumulation of lactate is a key component of glucose metabolism. PDK1 has been shown to be a direct target gene of HIF-1 [11,27]. Hypoxia induces HIF-1α to bind to the promoter region of PDK1, phosphorylate and inactivate the catalytic subunit of PDH, inhibit pyruvate oxidation in mitochondria, and switch to the formation of large amounts of lactic acid via fermentation in the cytoplasm [28]. In this experiment, we found that glucose consumption and lactate production in the culture medium of PASMCs from yellow cattles and yaks were significantly higher (*p* < 0.05) than those from yaks under hypoxic conditions. The differences in glucose consumption and lactate production between yak PASMCs under prolonged hypoxic conditions and yellow cattle and yak PASMCs under normoxic conditions were not significant (*p* > 0.05). The increased glucose content in the culture medium of yellow cattle and yak PASMCs under short-term normoxic conditions may have been related to the initiation of intracellular gluconeogenesis. This indicates that, under hypoxic conditions, the cells of the yellow cattle increase their uptake of glucose, but the hypoxia leads to the inhibition of the tricarboxylic acid cycle, and glucose cannot provide enough energy and carbon for the cells through aerobic respiration; it can only be converted into lactic acid, which can be excreted from the cells.

Hypoxia induces HIF-1α to bind to the PDK1 promoter region, phosphorylating and, thus, inactivating the catalytic subunit of PDH, and HIF-1α is not degraded and translocates to the nucleus, where it forms an HIF-1 dimer with the HIF-1β subunit, which is then regulated by binding to the HRE and participates in the regulation of the expression of the target gene, PDK1 [11,27]. Hypoxic stimulation of cells also induces the accumulation and phosphorylation of Akt in mitochondria. p-Akt further promotes the phosphorylation of PDK1 in mitochondria and activates Akt-PDK1 signaling. By activating Akt-PDK1 signaling, p-PDK1 upregulates HIF-1α and promotes TGF-β/Smad signaling, and TGF-β activation contributes to the phosphorylation of Smad2/3 proteins [29,30]. At the same time, hypoxia induces TGF-β/Smad signaling to upregulate VEGF expression, promoting the proliferation of vascular smooth muscle and vascular endothelial cells (PAECs) and accelerating the process of vascular remodeling [31]. At the same time, hypoxia-induced inhibition of HIF-1α protein degradation causes HIF-1α to bind to hypoxia-responsive components of VEGF, resulting in an increase in VEGF expression [32,33]. There is a close relationship between HIF-1α, PDK1, and the TGF-β/Smad signaling pathways, but studies on the effects of changes in their expression under hypoxic conditions in yellow cattle and yak PASMCs have not been reported. At the gene level, it was found that the expression of the PDK1 and TGF-β/Smad pathway-related genes in yellow cattle PASMCs changed with the prolongation of hypoxia time compared with normoxia. Smad2 and Smad3 interact with each other, which in turn leads to less identical trends in Smad2 and Smad3 expression [34]. The experimental results elicited downstream SphK1 and Snail expression trends consistent with Smad2 and VEGF-A expression trends consistent with Smad3, but Smad2 and Smad3 were upregulated in expression, which in turn promoted cell proliferation and migration, and it was hypothesized that SphK1 and Snail were regulated by Smad2, and VEGF-A was regulated by Smad3; the high expression of Bcl-2 improves the antiapoptotic ability of yellow cattle PASMCs. In contrast, yak PASMCs were not significantly affected by changes in the relative expression of Akt1 and PDK1, etc. However, when considering mRNA with the duration of hypoxia, compared with normoxia, the relative expression of Akt1 and α-SMA mRNA was increased, and the relative expression of PDK1, HIF-1α, TGF-β1, Smad2, Smad3, VEGF-A, Snail, and Gal-3 mRNA was significantly decreased, which attenuated the effects of hypoxia on lung vascular remodeling induced by proliferation, migration, and anti-apoptosis of yak PASMCs. SphK phosphorylates sphingosine (Sph) to sphingosine-1-phosphate (S1P), which acts as a first messenger to G protein-coupled receptors (S1P Receptors, S1PRs) on the cell surface and plays a role in promoting cell proliferation, increasing angiogenesis and inhibiting apoptosis [35]. The relative expression of SphK1 mRNA showed a trend of decreasing and then increasing under hypoxic conditions, and it was ultimately close to that under normoxia; these results indicate that the yak PASMCs adapted to the effects of hypoxia on lung tissue by downregulating the expression of genes related to the PDK1 and TGF-β/Smad signaling pathway.

Currently, studies on the relationship between PDK1 and hypoxia regulation have focused on tumor- and cancer-cell-related studies. PDK1 phosphorylates the pyruvate dehydrogenase complex, thereby inhibiting its activity; inhibition of the pyruvate dehydrogenase complex inhibits the tricarboxylic acid cycle and metabolic reprogramming of tumor cells to glycolysis, and transcriptional and post-transcriptional control of the PDK family is one way in which cancer cells can alter normal pyruvate metabolism to promote proliferation [36]. It was found that the expression of HIF-1α mRNA and protein was significantly increased under hypoxic conditions compared to aerobic conditions; lactate production, the sugar-metabolizing enzyme PDK1, lactate dehydrogenase A (LDHA), pyruvate kinase M2 (PKM2) mRNA, and protein expression were significantly increase [37]. Meanwhile, PDK1 knockdown reduced the proliferation, migration, and tumorigenicity of breast cancer cells and inhibited the HIF-1α signaling pathway [36]. Studies have shown that PDK1 increases HIF-1α protein stability by phosphorylating HIF-1α Ser451 and promotes HIF-1α’s transcriptional activity by increasing HIF-1α’s binding to P300, and PDK1 and HIF-1α form a positive feedback loop to promote breast cancer progression. In vitro and in vivo studies on breast cancer have shown that PDK1 increases cells’ proliferation, migration, and invasion, as well as tumor growth and metastasis [38]. In addition, inhibition of PDK1 by DCA, a PDK1 inhibitor, decreased the glycolytic status and cell proliferation of MDA-MB-435R (435R) cells, a human melanoma cell model [39]. This suggests that PDK1 plays an important role in the process of glucose metabolism and in the control of various cellular phenotypes. Cultured under normoxic conditions, it was found that the proliferation level of yak PASMCs overexpressing PDK1 was significantly increased (*p* < 0.05), and the number of cells was significantly higher than that of the other groups on the fifth day; their apoptosis level (mean = 3.077%) was lower than that of the blank vector control group (mean = 5.725%), and their antiapoptosis index Bcl-2/Bax ratio was not significant compared with that of the blank vector group (*p* > 0.05); its migration level was significantly higher, and it had completely healed at about 48 h; its glucose consumption and lactate production were significantly higher than that of the blank vector control group; its relative expression of HIF-1α and PDK1 mRNA was significantly higher than that of the blank vector group (*p* < 0.05); and their relative expression of the contractile surface marker α-SMA mRNA was significantly lower than that of the blank vector group (*p* < 0.05). This suggests that overexpression of PDK1 induced an increase in proliferation, anti-apoptosis, migration, glucose consumption, and lactate production in PASMCs under normoxia–hypoxia.

It has been shown that a decrease in mitochondrial reactive oxygen species (mROS) inhibits the cellular redox state, resulting in a pseudohypoxia-like state [40]. Meanwhile, it has been pointed out that HIF-1α is aerobically activated when cells are in a pseudohypoxic state, and activated HIF-1α promotes the cellular switch to aerobic glycolysis by upregulating PDK1 and PDK2 [41], which in turn promotes cell proliferation. Therefore, we suggest that this may be related to the fact that high PDK1 expression inhibits the activity of PDH, allowing HIF-1α to be activated without being degraded. We hypothesize that overexpression of PDK1 induces a pseudo-hypoxic state in yak PASMCs under normoxic conditions, which, in turn, promotes cell proliferation and, ultimately, leads to vascular remodeling.

Combined with the expression of PDK1 and its TGF-β/Smad pathway-related genes under hypoxic conditions, it was found that the relative expression of PDK1, HIF-1α, Smad2, Smad3, Bcl-2, VEGF-A, SphK1, Snail, and Gal-3 mRNAs was altered in yak PASMCs overexpressing PDK1, and the short-term hypoxic effects were significant. After prolonged hypoxia, Smad2, Smad3, Bcl-2, and VEGF-A were not significantly different from the normoxic blank vector control group, and Akt1 and TGF-β1 did not show significant changes in relative gene expression under hypoxic conditions. The relative expression of the Akt1, TGF-β/Smad, and VEGF-A genes was not significant after prolonged hypoxia, which may have been influenced by the expression at the protein level, and changes in the relative expression of the PDK1, HIF-1α, SphK1, Snail, and Gal-3 genes. Studies have shown that Snail promotes cell survival and inhibits apoptosis. Snail can inhibithe expression of a number of apoptotic gene that are mediated by p53, via direct transcription of programmed cell death-related inhibitory genes [42]; Gal-3 regulates the Smad2/3 signaling pathway through protein interactions with TGF-β1, which, in turn, regulates the proliferation and migration of human PASMCs, thereby modulating the onset and progression of pulmonary hypertension [43]; TGF-β1 promotes S1P synthesis and secretion by promoting Smad2/3 phosphorylation and upregulating SphK1 expression, which, in turn, promotes Notch3 activation and, ultimately, PASMC proliferation [44]. It was hypothesized that PDK1-overexpressing yak PASMCs were affected by short-term and long-term hypoxia in terms of their expression of PDK1-mediated TGF-β/Smad signaling pathway genes, which ultimately led to the PDK1-overexpressing yak PASMCs exhibiting reduced migration levels, proliferation, anti-apoptosis, ROS production, glucose consumption, and lactate production under hypoxia, which, in turn, were detrimental to their adaptation to the long-term hypoxic environment. Short- and long-term hypoxia affected the expression of PDK1-mediated TGF-β/Smad signaling pathway genes in PDK1-overexpressing yak PASMCs for further studies.

**Figure 18:**
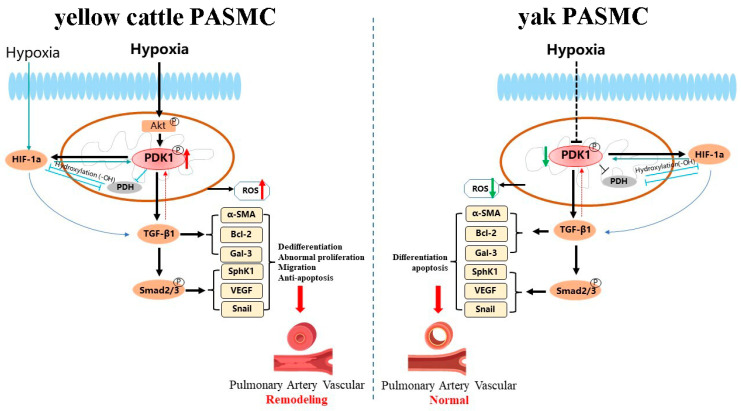
Regulation of PDK1-mediated TGF-β/Smad signaling pathway by hypoxia in yellow cattle and yak PASMCs. Solid lines indicate compatibility with the existing research base and experimental results on PDK1 and TGFβ/Smad signaling, and dashed lines indicate speculation with the experimental results that require further validation. Black is the pathway that is primarily affected by hypoxia in this study, and blue is the pathway that is secondarily affected by hypoxia.

In summary, combined with the effects of hypoxia on the phenotypic changes and the expression of PDK1 and its TGF-β/Smad pathway-related genes in yellow cattle and yak PASMCs, the following modes of PDK1 and TGF-β/Smad pathway regulation were hypothesized for hypoxia-induced pulmonary arterial hypertension (Figure 18): (1) Hypoxia may bind HIF-1α to PDK1, causing loss of PHDs activity and increased expression of HIF-1α without degradation, which in turn affects PDK1 expression. (2) Hypoxia may induce the expression of Akt1 in the mitochondria of yellow cattle PASMCs by stimulating their mitochondria, which in turn activates the expression of PDK1, which in turn promotes HIF-1α and TGF-β/Smad signaling, and the increased expression of TGF-β1 induces the upregulation of the expression of its downstream target genes such as Smad3, and the upregulation of the expression of Smad2/3 activates the expression of its downstream genes. (3) Conversely, compared with normoxia, hypoxia-induced PDK1 expression may be reduced in yak PASMCs, resulting in attenuated inhibition of PHDs, which in turn reduces the expression of HIF-1α; at the same time, the reduction in PDK1 expression also affects the changes in TGF-β/Smad downstream gene expression. As only cellular level studies have been conducted so far, the specific regulatory mechanisms of hypoxia on PDK1-mediated TGF-β/Smad signaling in yak lungs need to be further explored and investigated.

## 5. Conclusions

In conclusion, the present study is the first to focus on the differential regulation of lung adaptation to hypoxia in yellow cattle and yak around PDK1-mediated TGF-β/Smad signaling of yak lung hypoxia-adaptation-related genes screened by previous experimental work [33,45]. Compared with normoxia, hypoxia induced a possible reduction in PDK1 expression in yak PASMCs, and the inhibition of PHDs by PDK1 was attenuated, which in turn reduced the expression of HIF-1α; meanwhile, the reduction in PDK1 expression also affected the changes in TGF-β/Smad downstream gene expression. Short-term long-term hypoxia affects the expression of PDK1-mediated TGF-β/Smad signaling pathway gene levels in overexpressing PDK1-yak PASMCs. As for further studies, it is suggested that the regulation of PDK1-mediated TGF-β/Smad signaling may be involved in the process of yak adaptation to the hypoxic environment of the plateau, reflecting the yak’s own good adaptive ability. The present study provides basic information to further elucidate the mechanism of PDK1-mediated TGF-β/Smad signaling in the lungs of yaks induced by hypoxia, and, at the same time, provides target genes for the prevention and treatment of plateau diseases in humans and animals.

## Figures and Tables

**Table 1:** Lentiviral transfection solution preparation.

Component Name	System (μL)
virus volume	(MOI × cell number)/viral titer
HitranG P Infection Enhancement Solution	20
complete medium (10% FBS DME/F12)	-
Total	500

## Data Availability

All data presented in this study are available on request from the corresponding authors.

## Supplementary Materials

The following supporting information can be downloaded at: https://www.mdpi.com/article/10.3390/ani14162422/s1, Table S1: Primer sequence information.
