# Supplementary material for: Study on the Regulatory Mechanism of the PDK1-Mediated TGF-β/Smad Signaling Pathway in Hypoxia-Induced Yak Lungs
Source: Animals (Basel). 2024 Aug 21;14(16):2422. doi: 10.3390/ani14162422 (PMC11350703; doi:10.3390/ani14162422)
Supplement: Supplementary file 1 [file animals-14-02422-s001.zip › animals-3130805-supplementary.pdf]

Supplementary data

Synthesis of gene primers

According to the sequences of *TGF-β1*, *Smad2*, *Smad3*, *VEGF-A*, *Gal-3*, *SphK1*, *Snail*, *Bcl-2*, *Bax*, *α-SMA*, *Caspase3, PDK1* and *HIF-1α* genes in GenBank, Primer-BLAST was used to design the primers for these genes, at the same time, *β-actin* (*actin beta*, *ACTB*) gene was used as the internal reference gene. The information regarding the primer sequences is shown in Table S1.The data were sent to Hunan Accurate Bioengineering Co. (Hunan, China) for synthesis

Table S1. Primer sequence information

Primer	Primer sequence(5'~3')	Amplified fragment size/bp
TGF-β1	F: GCGGACTACTACGCCAAGGA<br>R: GCTGTGCGAGCTAGACTTCATTT	90
Smad2	F: TGCTGGCTCAGTCCGTTAAT<br>R: TTGTTACCGTCTGCCTTCGG	120
Smad3	F: GGGTGGATTTGGGGAAGAG<br>R: GGTTTGCTTTCGTGTTTTGG	132
VEGF-A	F: GGGGCTGCTGTAATGACGA<br>R: CCTATGTGCTGGCTTTGGTGA	104
Gal-3	F: CCCCTCCAAGATCCGAGAT<br>R: GTTCGCATTGGGCTTTACTG	81
SphK1	F: AGTATGGCTCAAAGCTGCCC<br>R: CAGCCAGGACGGTCAAAGT	89
Snail	F: ATGTCAAGAAGTACCAGTGCAAGA<br>R: AAGGAGCCTCAGGAGTCAGC	116
Bcl-2	F: GATGACCGAGTACCTGAACCG<br>R: GACAGCCAGGAGAAATCAAACA	120
Bax	F: CCTTTTGCTTCAGGGTTTCAT<br>R: CGCTCAGCTTCTTGGTGGAT	110
α-SMA	F: CAATGGCTCTGGGCTCTGT<br>R: CCTCTTTTGCTTTGTGCTTCA	156
PDK1	F: GCAAAATCACCAGGACAGCC<br>R: CGGATAAACACCTTTGTCAGCAT	129
Akt1	F: CCTAAAGAAGGAGGTCATCGTG<br>R: GGGACAGGTGGAAGAAAAGC	182
Caspase3	F: TGTCAAACAACAGCAATGACGA<br>R: CAGCACAAACATCACAAAACCA	149
HIF-1α	F: GGCGCGAACGACAAGAAAAA<br>R: GTGGCAACTGATGAGCAAGC	121
VEGF	F: CTGCTGTGGACTTGAGTTGGG<br>R: GCTGCCGTAAGAGGGATAAAA	107

β-actin (ACTB)	F: TCATCACCATCGGCAATGAG<br>R: AGCACCGTGTTGGCGTAGAG	157
